# Dynamic nomogram for predicting generalized conversion in adult-onset ocular myasthenia gravis

**DOI:** 10.1007/s10072-022-06519-5

**Published:** 2022-12-05

**Authors:** Zhuajin Bi, Yayun Cao, Mengcui Gui, Jing Lin, Qing Zhang, Yue Li, Suqiong Ji, Bitao Bu

**Affiliations:** 1 Department of Neurology, Tongji Hospital, Tongji Medical College, Huazhong University of Science and Technology, Wuhan, Hubei China; 2 Department of Radiology, Zhongnan Hospital of Wuhan University, Wuhan, Hubei Province China

**Keywords:** Adults, Generalization, Ocular myasthenia gravis, Predictors, Nomogram

## Abstract

**Purpose:**

To explore the factors and risk mapping model of progression from ocular myasthenia gravis (OMG) to generalized myasthenia gravis (GMG) in adult-onset patients.

**Methods:**

A retrospective, observational cohort study was performed for 435 OMG patients with onset age older than 14 years old. Multivariate Cox regression was used to identify the independent factors affecting generalized conversions that then were incorporated into the construction of the nomogram.

**Results:**

Two hundred thirty-seven patients (54.5%) had transformed into GMG after a median of 1.1 years (range 0.1-–9.1 years). The 6-, 12-, and 24-month generalized conversion rates were 31.7%, 49.8%, and 65.4%, respectively. Multivariable analysis showed that the early-onset age, male sex, concomitant autoimmune diseases (AID), positive results of anti-acetylcholine receptor antibodies, repetitive nerve stimulation abnormalities, the presence of thymoma, and prednisone treatment were significantly associated with the generalized conversions (hazard ratio [HR] = 0.598, 0.686, 1.554, 1.541, 2.020, 2.510, and 0.556, respectively). A nomogram was established to predict the possibility of generalization-free survival (GFS) in adult-onset OMG patients, and the model demonstrated good predictive performance with a C-index of 0.736 (95% confidence interval 0.703 ~ 0.769). Moreover, subgroup analyses were performed based on the presence or absence of prednisone therapy, and the results indicated that prednisone therapy has better prevention of generalized conversions in male, non-thymoma patients, and patients without other AID.

**Conclusion:**

A new predictive nomograph and web-based survival calculator we developed show favorable applicability and accuracy in predicting long-term GFS in adult-onset OMG patients.

**Supplementary Information:**

The online version contains supplementary material available at 10.1007/s10072-022-06519-5.

## Introduction

Myasthenia gravis (MG) is an immune-mediated neuromuscular disorder that is primarily caused by anti-acetylcholine receptor antibodies (AChR-ab), leading to fluctuating and fatigable muscle weakness [1]. MG is classified as ocular MG (OMG) and generalized MG (GMG) according to initial clinical symptoms [2]. The majority of patients with MG firstly present with ocular symptoms [3–5]. However, about 50 to 85% of OMG patients will progress to develop generalized muscular weakness, resulting in a poor prognosis [2, 4, 6]. Previously, many studies have demonstrated that age of onset can serve as a clinical factor affecting the prognosis of OMG patients, with a younger age being better than older age [4, 7, 8]. And the time to generalization was earlier in patients with adult-onset age than in those with juvenile-onset age [2]. These results suggested that it would be of great value to identify clinical factors associated with generalized conversions and to make individual assessments in OMG patients with adult-onset age. Nomograms have been used as a predictive model for estimating the probability of clinical outcomes, with relatively improved accuracy compared to traditional prognostic grouping or scoring systems [9]. In this model, clinically relevant variables are generally selected using multivariate analysis before model development and then assigned differential weights to facilitate individualized risk estimation. Herein, we try to establish the first nomogram to our knowledge for predicting conversion to generalization in adult-onset OMG patients in this study.

## Materials and methods

### Patient selection and study design

This retrospective cohort study was performed for patients with OMG who were treated at Tongji Hospital of Tongji Medical College, Huazhong University of Science and Technology from January 1989 to May 2020. The inclusion criteria were as follows: (1) patients with OMG were diagnosed according to the fatigable weakness limited to the extraocular muscles within the first month of onset and at least one of the following results: (a) response to the neostigmine test, (b) decremental response on slow-frequency repetitive nerve stimulation (RNS) test, or (c) abnormal increase of serum AChR-ab levels [10]; (2) age of onset was more than 14 years old. Patients were excluded if they had any of the following conditions: (1) patients concurrently presented with generalized symptoms within the first month of diseases, such as limb weakness, dysphagia, dysarthria, and even respiratory difficulties [2]; (2) extraocular muscle palsy was caused by other diseases, such as thyroid ophthalmopathy, cranial neuropathies, and mitochondrial myopathy [5]; (3) follow-up was less than 2 years; or (4) there was insufficient baseline data.

To investigate factors that may potentially affect the generalized conversion, which was defined as the appearance of any generalized symptoms beyond extraocular muscle weakness throughout our study, the patients were classified into two groups: those whose symptoms remained ocular (OMG-R) and those whose symptoms generalized (OMG-G).

### Data collection and therapy

The following patient characteristics were collected from medical records and face-to-face interviews: age at onset, gender, clinical symptoms at presentation, disease duration, progression time to GMG (if this occurred), thyroid dysfunction examination, concomitant autoimmune disease (AID), response to neostigmine and RNS tests, the serum status of AChR-ab estimated by radioimmunoassay (RIA) or enzyme-linked immunosorbent assay (ELISA) (RSR Limited, Cardiff, UK), thymic abnormalities based on chest computed tomography (CT) or histological examination, pharmaceutical and thymectomy treatments, Myasthenia Gravis Foundation of America (MGFA) classification [11], and MGFA post-intervention status (PIS) at the last visit [11].

All patients were firstly treated with pyridostigmine once diagnosed with OMG. Prednisone was given to those patients who had not improved satisfactorily after sufficient pyridostigmine therapy for 2–4 weeks [12]. The initial dose of prednisone was 10 mg and was increased by increments of 10 mg every 2 days up to 1.0 mg/kg of body weight, and then was gradually reduced by 5–10 mg per month after a noticeable improvement of symptoms. Besides, the total course of prednisone treatment lasted more than 6 months. Nonsteroidal immunosuppressant (IS) therapy was excluded from multivariate analysis, considering that fewer subjects (12.9%) received IS treatment and no significant difference in IS treatment was found between the OMG-R and OMG-G groups (*P* = 0.425). Furthermore, the enrolled patients were divided into two groups based on the presence or absence of prednisone treatment: the prednisone-pyridostigmine group and the pyridostigmine group. Thymectomy would be an optional method for patients with thymoma or those unresponsive to pharmacologic therapies [13].

### Statistical analysis

Numerical data are presented as mean ± standard deviation (SD) or median (interquartile range, IQR), and categorical data are presented as frequencies with absolute numbers and percentages. The missing data of AChR-ab and RNS tests account for less than 30% and were supplemented by multiple interpolations in the mice package of R software [14]. Correlations between clinical factors were evaluated using Spearman’s rank correlations. By using the Cox models, subgroup analyses are performed for clinical factors associated with generalized conversion rates in patients with or without prednisone treatment. Survival curves were calculated using the Kaplan–Meier curves and compared using the log-rank test. A univariate Cox regression analysis was applied to identify possible factors correlated with the development of GMG and entered variables with *P* value < 0.20 into the multivariate Cox regression analysis. According to the results of multivariate Cox regression analysis, a nomogram was formulated by using the rms package of R software. Subsequently, the nomograms were transformed into convenient online versions by using the shinyPredict package of R software. Harrell’s concordance index (C-index), receiver operating characteristic curve (ROC), and the area under the ROC curve (AUC) were used to assess discrimination of the model, while the calibration plot was used to graphically evaluate the calibration of the model based on internal validation with 1000 bootstrap samples [9]. All data analyses were performed with R version 4.0.4 and a two-tailed *P* < 0.05 was deemed to indicate statistical significance.

## Results

### Demographic characteristics

**Fig. 1:**
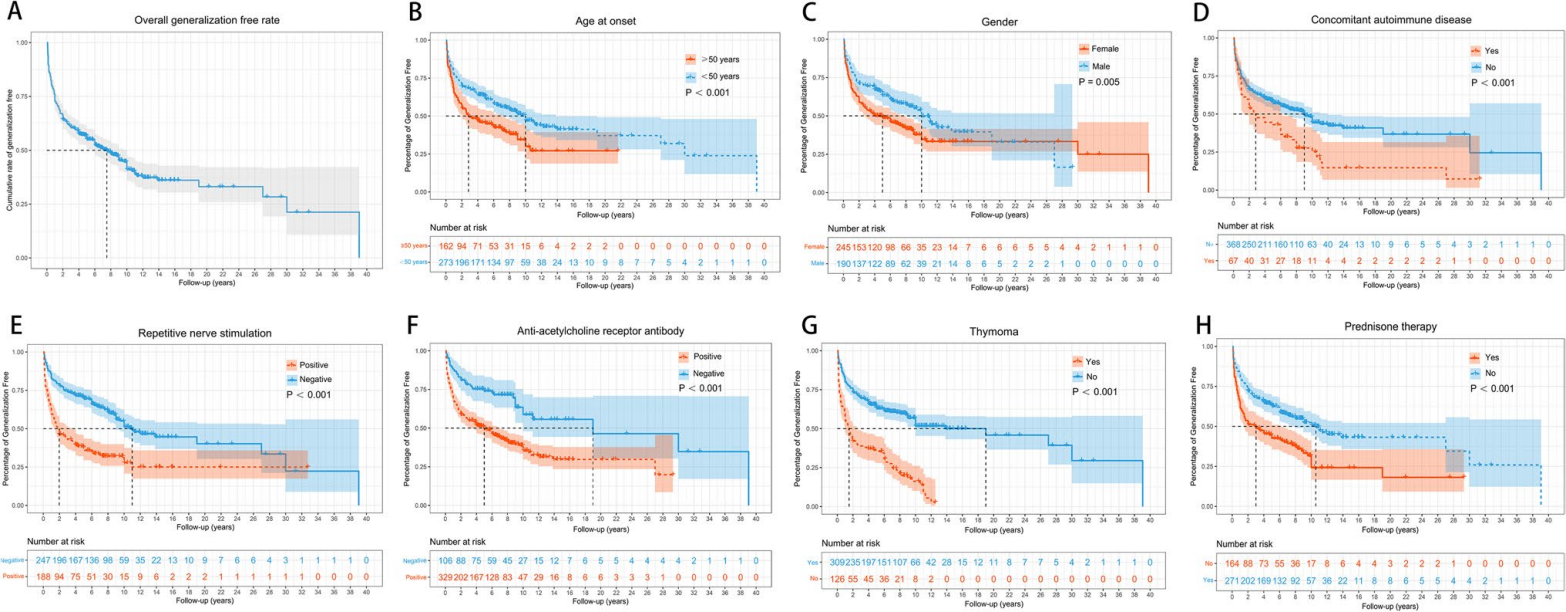
The Kaplan–Meier curve for time to generalized conversion in adults with ocular myasthenia gravis during the entire study period. **A** Cumulative rate of generalization free in all patients, the median time of generalized conversion in all patients was 7.5 years. **B** Age of onset, the median time of generalized conversion in patients aged ≥ 50 years and < 50 was 2.8 and 10.0 years, respectively (*P* < 0.001). **C** Gender, the median time of generalized conversion in male and female patients was 10.0 years and 5.0 years, respectively (*P* = 0.005). **D** Concomitant autoimmune diseases, the median time of generalized conversion in patients with and without concomitant autoimmune diseases was 2.8 years and 9.0 years, respectively (*P* < 0.001). **E** Repetitive nerve stimulation, the median time of generalized conversion in positive and negative patients was 1.9 years and 11.0 years, respectively (*P* < 0.001). **F** Anti-acetylcholine receptor antibodies, the median time of generalized conversion in positive and negative patients was 5.0 years and 19.0 years, respectively (*P* < 0.001). **G** Thymoma, the median time of generalized conversion in patients with and without thymoma was 1.5 years and 19.0 years, respectively (*P* < 0.001). **H** Prednisone therapy, the median time of generalized conversion in patients with and without prednisone therapy was 10.6 years and 3.0 years, respectively (*P* < 0.001)

A total of 435 patients (median [IQR] age at onset: 44.1 [29.3, 54.9] years; 56.3% female) were enrolled in the study. The basic characteristics of enrolled patients are shown in Table 1. The percentage of early-onset patients (< 50 years) was 62.8% and of late-onset patients (≥ 50 years) was 37.2%. Compared with male patients, female patients had a younger age of onset, longer disease duration, more concomitant AID and thymic hyperplasia, and more severe MGFA classification (all *P* < 0.05) (seen in Supplementary Table S1). The median duration of disease from ocular symptom onset to the last follow-up was 7.8 years (IQR: 4.0, 10.7). The positive AChR-ab or RNS was detected in 329 (75.6%) patients and 134 (40.6%) patients, respectively. Thymus status was evaluated in all patients by chest CT scan or thymus pathology, including 126 thymomas, 75 thymus hyperplasia, and 234 normal thymuses. Ptosis was the most common initial presentation in 245 patients (56.3%). More importantly, 237 adult-onset patients with OMG (54.5%) progressed to GMG after a median of 1.1 years (IQR: 0.3, 4.0) (range 0.1–39.1 years). And 31.7% of generalized conversions occurred in the 6 months of onset, 49.8% in the first year, and 65.4% in the first 2 years (Fig. 1A). Among the 237 patients undergoing generalization, 89 (37.6%) patients progressed to type IIA and 96 (40.5%) patients progressed to type IIB according to the MGFA classification. Thymectomy had been performed in 104 patients (23.9%) before the development of generalization and the median (IQR) time from onset to thymectomy was 2.8 months (1.0, 13.0). The maximum clinical severity of patients was classified as ocular MG (MGFA I) in 198 patients (45.5%), mild (MGFA II) in 140 patients (32.2%), and moderate to severe (MGFA III-V) in 97 patients (22.3%). In the last follow-up visit, 263 cases (79.0%) attained MMS or better status, but 77 cases (17.7%) did not improve or worsen.

**Table 1:** Baseline characteristics of 435 study participants

Characteristics	Patients
Gender	
Male	190 (43.7)
Female	245 (56.3)
Age at onset, y^a^	44.1 (29.3, 54.9)
EOMG (< 50 y)	273 (62.8)
LOMG (≥ 50 y)	162 (37.2)
Disease duration, y	7.8 (4.0, 10.7)
Initial symptoms	
Ptosis	245 (56.3)
Diplopia	38 (8.7)
Ptosis with diplopia	152 (35.0)
Thyroid dysfunction^b^	72 (16.6)
Concomitant AID	67 (15.4)
Neostigmine test ( +)	391 (89.9)
RNS ( +)	134/330
AChR-ab ( +)^c^	271/334
Thymus type^d^	
Normal	234 (53.8)
Hyperplasia	75 (17.2)
Thymoma	126 (29.0)
Pre therapy	271 (62.3)
Pre + IS therapy	56 (12.9)
Thymectomy	104 (23.9)
Time from onset to thymectomy, m	2.8 (1.0, 13.0)
Age at thymectomy, y	43.2 (32.4, 52.1)
MGFA classification at generalization^e^	
IIA/IIB	89/96
IIIA/IIIB	12/16
IVA/IVB	9/7
V	8
MGFA classification (most severe)	
I	198
IIA/IIB	66/74
IIIA/IIB	14/25
IVA/IVB	8/12
V	38
MGFA-PIS at last visit	
CSR	54 (12.4)
PR	40 (9.2)
MMS	169 (38.8)
Improved	95 (21.8)
Unchanged	13 (3.0)
Worse	19 (4.4)
Exacerbated	39 (9.0)
Dead	6 (1.4)

Data are given as *n* (%) or median (interquartile range)

^a^As for age of onset, the patients can be divided in early-onset (EOMG) and late-onset (LOMG) with onset before or after 50 years

^b^Thyroid dysfunction included 43 hyperthyroidism, 16 subclinical hyperthyroidism, 5 hypothyroidism, and 8 subclinical hypothyroidism

^c^The AChR-ab titers > 0.50 nmol/L were defined as positive (ELISA or RIA kit, RSR Limited, Cardiff, UK)

^d^Thymus status was evaluated by chest computed tomography (CT) scan in non-thymectomized patients and thymus histology in thymectomized patients

^e^The denominators are the number of patients with generalization throughout the study

Abbreviations: *AChR-ab*, anti-acetylcholine receptor antibodies; *AID*, autoimmune disease; *CSR*, complete stable remission; *EOMG*, early-onset myasthenia gravis; *IS*, immunosuppressants; *LOMG*, late-onset myasthenia gravis; *MG*, myasthenia gravis; *MGFA*, Myasthenia Gravis Foundation of America; *MMS*, minimal manifestation status; *PIS*, post-intervention status; *PR*, pharmacologic remission; *Pre*, prednisone; *RNS*, repetitive nerve stimulation

### Kaplan–Meier curve and subgroup analysis

**Fig. 2:**
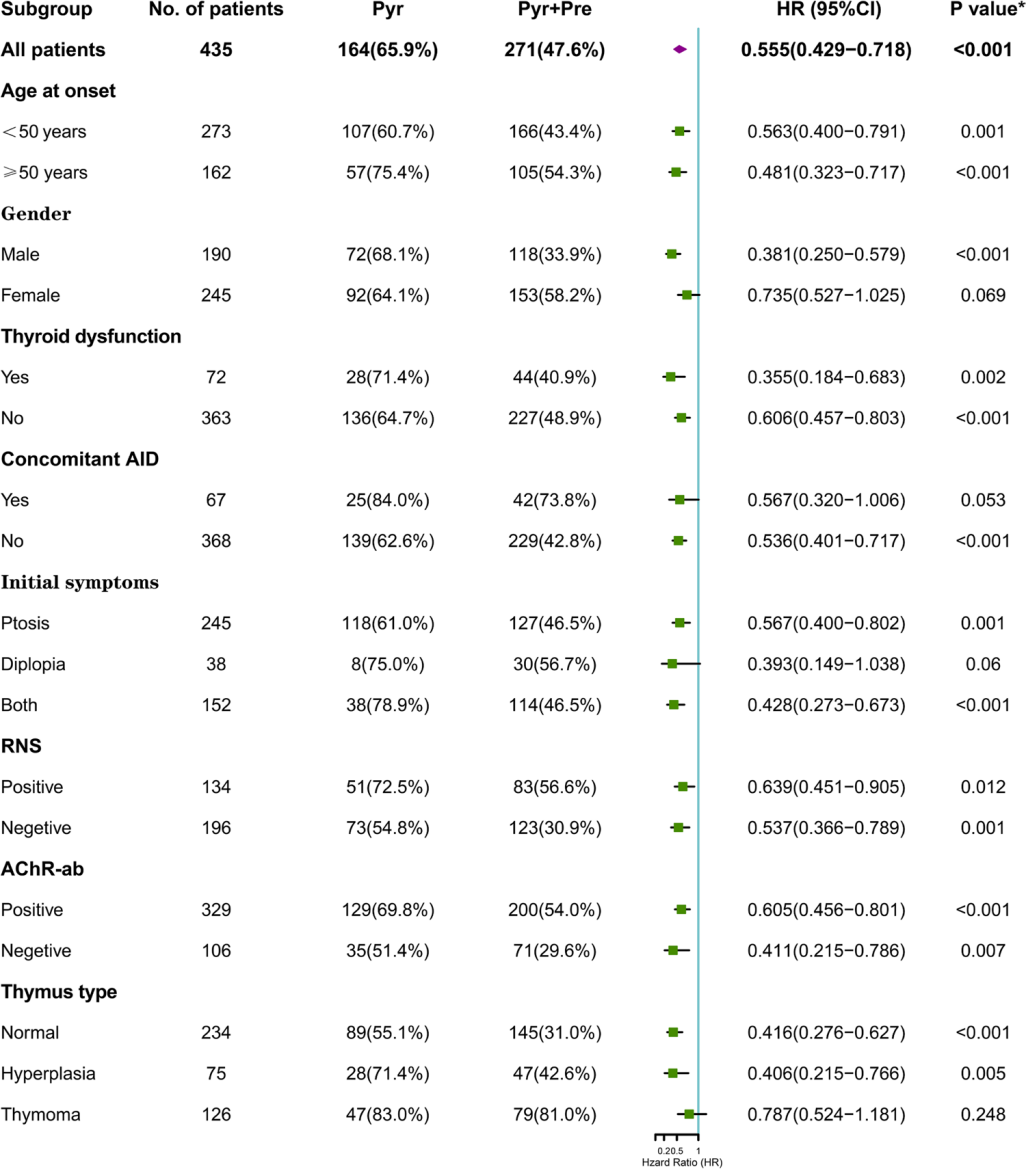
Univariable Cox regression analysis and forest plot of potential predictors for generalized conversion in patients with or without prednisone treatment. *P value for interaction of treatment and the factor. There were significant interactions in gender, presence or absence of concomitant AID or thymoma (*P* < 0.05). Abbreviations: AChR-ab, anti-acetylcholine receptor antibodies; AID, autoimmune disease; CI, confidence interval; HR, hazard ratio; Pre, prednisone; Pyr, pyridostigmine; RNS, repetitive nerve stimulation

The Kaplan–Meier method was used to obtain cumulative probabilities of generalization-free survival (GFS) by stratifying patients according to age at onset (Fig. 1B), gender (Fig. 1C), concomitant AID (Fig. 1D), RNS results (Fig. 1E), AChR-ab status (Fig. 1F), the presence of thymoma (Fig. 1G), and the use of prednisone therapy (Fig. 1H). The analysis demonstrated that patients with early-onset age, concomitant AID, positive AChR-ab or RNS, thymoma, pyridostigmine treatment alone, and female patients had higher conversion rates and earlier time to generalization than those with late-onset age, non-concomitant AID, negative AChR-ab or RNS, non-thymoma, prednisone treatment, and male patients (all *P* < 0.05). Furthermore, subgroup analysis was performed based on the presence or absence of prednisone treatment, and the results showed that the generalized conversion rate was significantly lower in males, patients without additional AID, and non-thymoma patients in the prednisone-pyridostigmine group than that in the pyridostigmine group (all *P* < 0.05) (Fig. 2).

### Factors for conversion to generalization

The patients were divided into two groups: the OMG-R group (*n* = 198) and the OMG-G group (*n* = 237). The common clinical features of the two groups were available in Supplementary Table S2. Age at onset (< 50 years) (HR = 0.631, 95%CI 0.457 ~ 0.782;* P* = 0.001), male sex (HR = 0.689, 95%CI 0.486 ~ 0.819;* P* = 0.006), disease duration (HR = 1.018, 95%CI 0.991 ~ 1.045;* P* = 0.194), concomitant AID (HR = 1.694, 95%CI 1.245 ~ 2.306; *P* < 0.001), RNS abnormalities (HR = 2.297, 95%CI 1.773 ~ 2.976;* P* < 0.001), positive AChR-ab (HR = 2.252, 95%CI 1.584 ~ 3.202; *P* < 0.001), thymoma (HR = 2.931, 95%CI 2.256 ~ 3.809;* P* < 0.001), prednisone therapy (HR = 0.555, 95%CI 0.429 ~ 0.718;* P* < 0.001), and thymectomy (HR = 1.635, 95%CI 1.239 ~ 2.159;* P* < 0.001) were found to be associated with the generalized conversions using univariate cox regression analysis (Table 2). Further multivariate cox regression analysis revealed that concomitant AID (HR = 1.554, 95%CI 1.128 ~ 2.141;* P* = 0.007), RNS abnormalities (HR = 2.020, 95%CI 1.549 ~ 2.632;* P* < 0.001), positive AChR-ab (HR = 1.541, 95%CI 1.070 ~ 2.220;* P* = 0.020), and thymoma (HR = 2.510, 95%CI 1.920 ~ 3.281;* P* < 0.001) were independent risk factors for the development of generalized myasthenia; while onset age < 50 years (HR = 0.598, 95%CI 0.457 ~ 0.782;* P* < 0.001), male sex (HR = 0.686, 95%CI 0.523 ~ 0.901;* P* = 0.007), and prednisone therapy (HR = 0.556, 95%CI 0.427 ~ 0.723;* P* < 0.001) predicted the reduction of the risk of generalized conversions.

**Table 2:** Univariate and multivariate Cox regression analysis of risk factors for generalization in the cohort of 435 patients

Variable	Univariable		Multivariable	
	HR (95% CI)	*P* value	HR (95% CI)	*P* value
Age at onset (< 50 y)	0.631 (0.486, 0.819)	0.001**	0.598 (0.457, 0.782)	< 0.001**
Gender (male)	0.689 (0.529, 0.897)	0.006**	0.686 (0.523, 0.901)	0.007**
Duration, y	1.018 (0.991, 1.045)	0.194*		
Symptoms at onset				
Ptosis	0.854 (0.660, 1.105)	0.229		
Diplopia	1.243 (0.801, 1.930)	0.332		
Both	1.101 (0.843, 1.439)	0.480		
Thyroid dysfunction	1.004 (0.705, 1.429)	0.984		
Concomitant AID	1.694 (1.245, 2.306)	< 0.001**	1.554 (1.128, 2.141)	0.007**
Neostigmine test ( +)	0.914 (0.606, 1.378)	0.357		
RNS ( +)	2.297 (1.773, 2.976)	< 0.001**	2.020 (1.549, 2.632)	< 0.001**
AChR-ab ( +)	2.252 (1.584, 3.202)	< 0.001**	1.541 (1.070, 2.220)	0.020**
Thymus abnormalities				
Hyperplasia	1.079 (0.767, 1.516)	0.663		
Thymoma	2.931 (2.256, 3.809)	< 0.001**	2.510 (1.920, 3.281)	< 0.001**
Pre therapy	0.555 (0.429, 0.718)	< 0.001**	0.556 (0.427, 0.723)	< 0.001**
Thymectomy	1.635 (1.239, 2.159)	< 0.001**		

^*^p < 0.2; **p < 0.05

Abbreviations: *AChR-ab*, anti-acetylcholine receptor antibodies; *AID*, autoimmune disease; *CI*, confidence interval; *HR*, hazard ratio; *Pre*, prednisone; *RNS*, repetitive nerve stimulation

### Nomogram development and validation

**Fig. 3:**
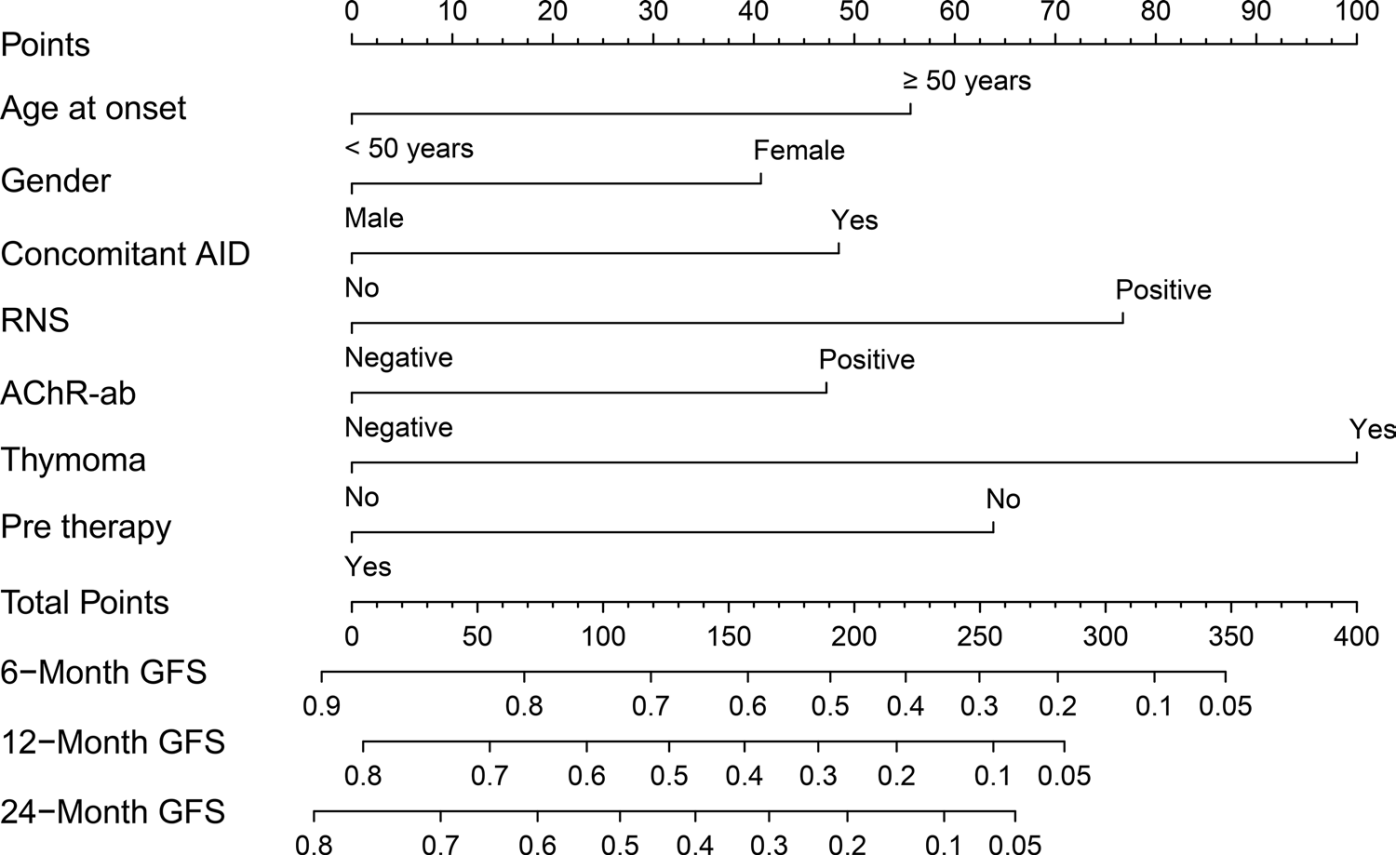
The nomogram model for predicting generalization in adults with OMG. To use the nomogram, find the position of each variable on the corresponding axis, draw a line to the points axis for the number of points, add the points from all of the variables, and draw a line from the total points axis to determine the GPS probabilities at the lower line of the nomogram. Abbreviations: AChR-ab, anti-acetylcholine receptor antibodies; AID, autoimmune disease; CI, confidence interval; GFR, generalization-free survival; Pre, prednisone; RNS, repetitive nerve stimulation

**Fig. 4:**
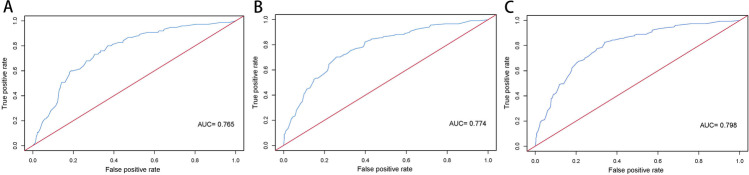
ROC curve and AUC of the nomogram for the prediction of 6-month GFS (**A**), 12-month GFS (**B**), and 24-month GFS (**C**)

**Fig. 5:**
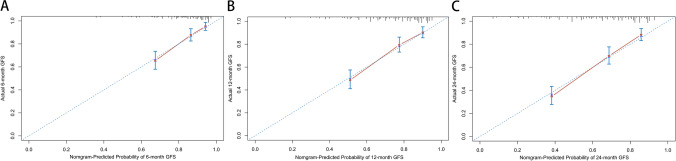
Calibration curves of the nomogram for the prediction of 6-, 12-, and 24-month GFS (**A**–**C**). The abscissa represents the nomogram-predicted GFS rate, the ordinate represents the actual GFS rate, and the calibration curves for 1-, 12-, and 24-month GFS rates showed satisfactory agreements between the predicted and actual values

**Fig. 6:**
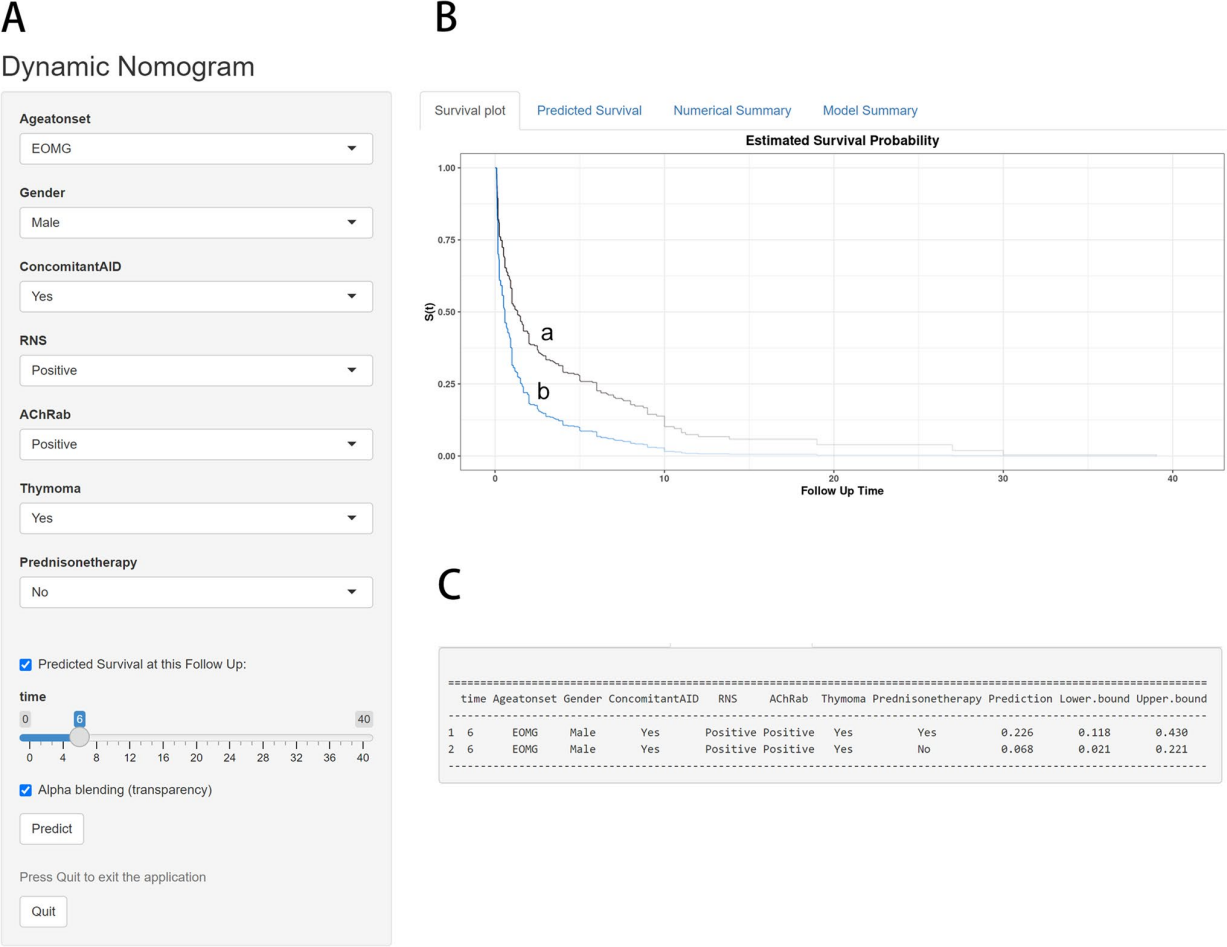
An example to illustrate the use of the web-based GFS rate calculator. **A** A 40-year-old male with OMG, who had combined with thymoma and other autoimmune diseases, tested positive for RNS and AChR-ab. **B** His generalization-free survival curve based on whether he received prednisone (a) or not (b). **C** The 6-month GFS rate was approximately 0.226 (95% CI 0.118–0.430) if he received prednisone treatment, whereas the 6-month GFS rate was approximately 0.068 (95% CI 0.021–0.221) if he did not receive prednisone treatment

The above 7 clinical predictive factors of generalized conversions were used to form a GFS estimation nomogram (Fig. 3). The performances of this nomogram were assessed by C-index, AUC, and calibration plots. To be specific, the value of the C-index and AUC ranges from 0.5 to 1.0, with 0.5 indicating random chance and 1.0 demonstrating perfect discrimination; the calibration curve showed that the closer each point is to the 45-degree line, the better the consistency between the predicted and actual probability. Our results showed that the C-index of the predictive model was 0.736 (95% CI 0.703 ~ 0.769). Subsequently, we drew the ROC curves of the nomogram for the prediction of 6-, 12- and 24-month GFS with the AUC value indicated (Fig. 4). The AUC for predicting 6-, 12-, and 24-month GFS was 0.765, 0.774, and 0.798, respectively, indicating good discrimination of the predictive model. The calibration plots also showed excellent agreement between the predicted probability of GFS and actual observation, which indicated favorable calibration of the model (Fig. 5). Finally, a dynamic web-based survival rate calculator based on the nomogram was established to predict long-term GFS (https://bizhuajin.shinyapps.io/DynNomapp/). For instance, a 40-year-old male with OMG, who had combined with thymoma and other AID, tested positive for RNS and AChR-ab. The 6-month GFS rate was approximately 0.226 (95% CI 0.118–0.430) if he received early prednisone therapy, whereas the 6-month GFS rate was approximately 0.068 (95% CI 0.021–0.221) if he did not receive early prednisone therapy (Fig. 6).

## Discussion

In this study, we performed a large retrospective cohort analysis of adult-onset OMG patients from our clinic to explore and establish a nomograph model for predicting generalized conversions. Our results showed more than half of patients converted to generalized myasthenia and the majority of conversions occurred within the first 2 years after symptom presentation, which was similar to previous studies [4, 15, 16]. And the nomogram indicated that generalized conversions were independently influenced by age at onset, gender, the results of AChR-ab or RNS tests, the presence of thymoma, and prednisone therapy.

The OMG patients usually present with unilateral or bilateral ptosis, diplopia, or both, with ptosis being the most common initial symptom [5, 8]. Wang et al. [4, 17] reported that the single initial symptom such as ptosis or diplopia predicted the early development of GMG than concurrence of ptosis and diplopia in OMG patients. However, our results showed no significant correlation between initial symptoms and generalized conversions. Previous studies indicated that gender seems to be an irrelevant factor for the generalization of OMG [1, 2, 7, 10]. The female patients in our cohort were more inclined to progress to GMG, which may be the result of a combination of sex hormones and genetic predisposition on the immunological function [5, 16]. Moreover, our findings confirmed a positive correlation between onset age and generalization (*r* = 0.200, *P* < 0.001), which was consistent with the analysis of Kupersmith [15]. Our results also demonstrated that late-onset age was an independent risk factor for the progression of GMG, suggesting an increased probability of worsening in patients’ onset after the age of 50 years old [16].

Accumulating evidence suggests that the abnormalities of the RNS test have been considered predictive factors for the generalization of OMG [10]. In the present study, the RNS test has relatively low sensitivity with a positive rate of 40.6%, and the positive results of the RNS test were correlated with a higher risk of generalization [5, 16]. Some scholars pointed out that electromyography was more sensitive to detecting the subclinical weakness of generalized muscles, and recommended that OMG patients with positive RNS results should be classified as latent general myasthenia gravis (LG-MG) [18, 19]. Our findings also suggested positive results of AChR-ab in the early stages of the disease could predict more generalized conversions, which was in line with the results of previous reports [2, 8, 10, 15]. In addition, Peeler et al. reported that higher AChR-ab levels at presentation were associated with an increased risk of developing generalized MG [1]. Another study suggested that anti-muscle specific kinase (MuSK) positive cases have a greater risk of generalization, in comparison with AChR-positive cases [20]. Unfortunately, we were unable to detect the levels of AChR-ab and MuSK-ab due to restrictions of examination methods, but these variables were strongly recommended to be included in future studies. It should be pointed out that the missing data from medical records resulted in an inadequate sample size for the RNS or AChR-ab test, due to the limitations of the retrospective study. Multiple interpolation method was used to supplement the missing data of these tests, which made the conclusions of this study more reliable [14].

Consistent with previous studies, our data indicated that the presence of thymoma has been identified as a risk factor for the generalization of OMG [10, 21]. Moreover, some researchers have investigated the encouraging role of thymectomy in reducing the risk of generalization [13, 22]. However, thymectomy was excluded as a potential predictor by multiple Cox regression analysis in the present study. This might be interpreted to some extent by the confounding factors of thymoma or prednisone therapy since they could eliminate or weaken the effect of thymectomy in preventing generalized conversions [23]. The effect of immunosuppressive therapy on the generalization of OMG remains controversial in literature [2, 10, 12, 24]. Our results demonstrated that prednisone therapy has protective effects on generalized conversions. We additionally performed subgroup analyses to determine the specific population who can benefit from prednisone treatment, and the results suggested that prednisone was more likely to play a protective role of generalization in males, non-thymoma patients, and patients without other AID. Given that this study adopted the principle of non-randomization treatment for enrolled patients, the predictive value of prednisone treatment and thymectomy needs to be further validated in long-term prospective randomized controlled trials [25].

By combining 7 clinical predictive factors of generalized conversions, a nomogram was constructed. The model provides an optimal estimation in predicting the probability of GFS in adults with OMG, with favorable accuracy and predictability.

## Supplementary Information

Below is the link to the electronic supplementary material.

Supplementary file1 (DOCX 28 KB)

## Data Availability

The data that support the findings of this study are available from the corresponding author upon reasonable request.

## References

[1] Peeler CE, De Lott LB, Nagia L (2015). Clinical utility of acetylcholine receptor antibody testing in ocular myasthenia gravis. JAMA Neurol.

[2] Ding J, Zhao S, Ren K (2020). Prediction of generalization of ocular myasthenia gravis under immunosuppressive therapy in Northwest China. BMC Neurol.

[3] Grob D, Brunner N, Namba T (2008). Lifetime course of myasthenia gravis. Muscle Nerve.

[4] Wang L, Zhang Y, He M (2017). Clinical predictors for the prognosis of myasthenia gravis. BMC Neurol.

[5] O'Hare M, Doughty C (2019). Update on ocular myasthenia gravis. Semin Neurol.

[6] Hehir MK, Silvestri NJ (2018). Generalized myasthenia gravis: classification, clinical presentation, natural history, and epidemiology. Neurol Clin.

[7] Mao ZF, Mo XA, Qin C (2010). Course and prognosis of myasthenia gravis: a systematic review. Eur J Neurol.

[8] Kamarajah SK, Sadalage G, Palmer J (2010). Ocular presentation of myasthenia gravis: a natural history cohort. Muscle Nerve.

[9] Steyerberg EW, Vergouwe Y (2014). Towards better clinical prediction models: seven steps for development and an ABCD for validation. Eur Heart J.

[10] Hong YH, Kwon SB, Kim BJ (2008). Korean Research Group for Neuromuscular Diseases. Prognosis of ocular myasthenia in Korea: a retrospective multicenter analysis of 202 patients. J Neurol Sci.

[11] Sanders DB, Wolfe GI, Benatar M (2016). International consensus guidance for management of myasthenia gravis: executive summary. Neurology.

[12] Cornblath WT (2018) Treatment of ocular myasthenia gravis. Asia Pac J Ophthalmol (Phila) 7(4):257–259. 10.22608/APO.2018301 10.22608/APO.2018301 30044061

[13] Kerty E, Elsais A, Argov Z (2014). EFNS/ENS Guidelines for the treatment of ocular myasthenia. Eur J Neurol.

[14] Li YM, Zhao P, Yang YH (2021) [Simulation study on missing data imputation methods for longitudinal data in cohort studies]. Zhonghua Liu Xing Bing Xue Za Zhi 42(10):1889–1894. Chinese. 10.3760/cma.j.cn112338-20201130-01363 10.3760/cma.j.cn112338-20201130-01363 34814629

[15] Kupersmith MJ, Latkany R, Homel P (2003). Development of generalized disease at 2 years in patients with ocular myasthenia gravis. Arch Neurol.

[16] Mazzoli M, Ariatti A, Valzania F (2018). Factors affecting outcome in ocular myasthenia gravis. Int J Neurosci.

[17] Wang LL, Zhang Y, He ML (2015). Clinical features and prognosis of ocular myasthenia gravis patients with different phenotypes. Chin Med J (Engl).

[18] Shinomiya N, Nomura Y, Segawa M (2004). A variant of childhood-onset myasthenia gravis: HLA typing and clinical characteristics in Japan. Clin Immunol.

[19] Plomp JJ, Huijbers MGM, Verschuuren JJGM (2018). Neuromuscular synapse electrophysiology in myasthenia gravis animal models. Ann N Y Acad Sci.

[20] Galassi G, Mazzoli M, Ariatti A (2018). Antibody profile may predict outcome in ocular myasthenia gravis. Acta Neurol Belg.

[21] Li F, Hotter B, Swierzy M (2018). Generalization after ocular onset in myasthenia gravis: a case series in Germany. J Neurol.

[22] Li H, Ruan Z, Gao F (2021). Thymectomy and risk of generalization in patients with ocular myasthenia gravis: a multicenter retrospective cohort study. Neurotherapeutics.

[23] Bokoliya SC, Patil SA (2019). Assessment of pre and post-thymectomy myasthenia gravis. Neurol Res.

[24] Benatar M, Mcdermott MP, Sanders DB (2016). Muscle Study Group (MSG). Efficacy of prednisone for the treatment of ocular myasthenia (EPITOME): a randomized, controlled trial. Muscle Nerve.

[25] Verma R, Wolfe GI, Kupersmith MJ (2021). Ocular myasthenia gravis - how effective is low dose prednisone long term?. J Neurol Sci.

