# Supplementary material for: Dynamic nomogram for predicting generalized conversion in adult-onset ocular myasthenia gravis
Source: Neurol Sci. 2022 Dec 5;44(4):1383–91. doi: 10.1007/s10072-022-06519-5 (PMC10023757; doi:10.1007/s10072-022-06519-5)
Supplement: Supplementary file 1 — Supplementary file1 (DOCX 28 KB) [file 10072_2022_6519_MOESM1_ESM.docx]

**Table S1**: The comparison of clinical features between male patients and female patients.

| Characteristics | Male (n=190) | Female (n=245) | *P* value |
| --- | --- | --- | --- |
| Age at onset  EOMG (< 50y)  LOMG (≥ 50y) | 108 (56.8)  82 (43.2) | 165 (67.3)  80 (32.7) | 0.025* |
| Disease duration^a^, y | 6.8 (3.5, 10.3) | 8.4 (4.2, 11.3) | 0.030* |
| Symptoms at onset  Ptosis  Diplopia  Both | 111 (58.4)  16 (8.4)  63 (33.2) | 134 (54.7)  22 (9.0)  89 (36.3) | 0.737 |
| Thyroid dysfunction | 26 (13.7) | 46 (18.8) | 0.156 |
| Concomitant AID | 15 (7.9) | 52 (21.2) | < 0.001* |
| Neostigmine test (+) | 173 (91.1) | 218 (89.0) | 0.477 |
| RNS (+) | 55/153 | 79/177 | 0.109 |
| AChR-ab (+) | 121/147 | 150/187 | 0.626 |
| Thymus abnormalities^a^  Hyperplasia  Thymoma | 23 (12.1)  50 (26.3) | 52 (21.2)  76 (31.0) | 0.013*  0.283 |
| Pre therapy | 118 (62.1) | 153 (62.4) | 0.942 |
| Pre + IS therapy | 30 (15.8) | 26 (10.6) | 0.110 |
| Thymectomy | 43 (22.6) | 61 (24.9) | 0.583 |
| Time from onset to thymectomy, m | 2.7 (1.0, 12.8) | 3.1 (1.0, 14.1) | 0.955 |
| MGFA classification (most severe)  I  II  III-V | 101 (53.2)  52 (27.4)  37 (19.5) | 97 (39.6)  88 (35.9)  60 (24.5) | 0.019* |
| MGFA-PIS at last visit  MMS or better  Improved  Unchanged or worse | 118 (62.1)  40 (21.1)  32 (16.8) | 145 (59.2)  55 (22.4)  45 (18.4) | 0.824 |

Note

Data are given as n (%) or median (interquartile range).

Analysis of continuous data was done with the Mann–Whitney U test. Analysis of dichotomous data was done with the χ2 test or Fisher's exact test as appropriate. * : *p*< 0.05.

^a^ The disease duration was defined as the interval from ocular symptom onset to the last follow-up

^b^ The AChR-ab titers >0.50 nmol/L were defined as positive (RIA kit, RSR Limited, Cardiff, UK).

^c^ Thymus status was evaluated by chest computed tomography (CT) scan in non-thymectomized patients and thymus histology in thymectomized patients.

Abbreviations: AChR-ab, anti-acetylcholine receptor antibodies; AID, autoimmune disease; CSR, complete stable remission; EOMG, early-onset myasthenia gravis; IS, immunosuppressants; LOMG, late-onset myasthenia gravis; MG, Myasthenia gravis; MGFA, Myasthenia Gravis Foundation of America; MMS, minimal manifestation status; PIS, post-intervention status; PR, pharmacologic remission; Pre, prednisone; RNS, repetitive nerve stimulation.

**Table S2**: The comparison of clinical features between the OMG-G group and the OMG-R group.

| Characteristics | OMG-G group  (n=237) | OMG-R group  (n=198) | *P* value |
| --- | --- | --- | --- |
| Gender  Male  Female | 89 (37.6)  148 (62.4) | 101 (51.0)  97 (49.0) | 0.005* |
| Age at onset, y  EOMG (< 50y)  LOMG (≥ 50y) | 137 (57.8)  100 (42.2) | 136 (68.7)  62 (31.3) | 0.019* |
| Disease duration^a^, y | 7.9 (3.8, 11.1) | 7.3 (4.3, 10.3) | 0.227 |
| Symptoms at onset  Ptosis  Diplopia  Both | 131 (55.3)  23 (9.7)  83 (35.0) | 114 (57.6)  15 (7.6)  69 (34.8) | 0.718 |
| Thyroid dysfunction | 38 (16.0) | 34 (17.2) | 0.750 |
| Concomitant AID | 52 (21.9) | 15 (7.6) | < 0.001* |
| Neostigmine test (+) | 209 (88.2) | 182 (91.9) | 0.296 |
| RNS (+) | 84/162 | 50/168 | < 0.001* |
| AChR-ab (+)^b^ | 163/182 | 108/152 | < 0.001* |
| Thymus abnormalities^c^  Hyperplasia  Thymoma | 40 (16.9)  103 (43.5) | 35 (17.7)  23 (11.6) | < 0.001* |
| Pre therapy | 129 (54.4) | 142 (71.7) | < 0.001* |
| Pre + IS therapy | 24 (10.1) | 32 (16.2) | 0.425 |
| Thymectomy | 72 (30.4) | 32 (16.2) | < 0.001* |
| Time from onset to thymectomy, m | 2.8 (1.0, 14.5) | 3.2 (1.0, 12.5) | 0.773 |
| MGFA-PIS at last visit  MMS or better  Improved  Unchanged or worse | 93 (39.2)  80 (33.8)  64 (27.0) | 170 (85.9)  15 (7.6)  13 (6.6) | < 0.001* |

Note

Data are given as n (%) or median (interquartile range).

Analysis of continuous data was done with the Mann–Whitney U test. Analysis of dichotomous data was done with the χ2 test or Fisher's exact test as appropriate. * : *p*< 0.05.

^a^ The disease duration was defined as the interval from ocular symptom onset to the last follow-up

^b^ The AChR-ab titers >0.50 nmol/L were defined as positive (RIA kit, RSR Limited, Cardiff, UK).

^c^ Thymus status was evaluated by chest computed tomography (CT) scan in non-thymectomized patients and thymus histology in thymectomized patients.

Abbreviations: AChR-ab, anti-acetylcholine receptor antibodies; AID, autoimmune disease; CSR, complete stable remission; EOMG, early-onset myasthenia gravis; IS, immunosuppressants; LOMG, late-onset myasthenia gravis; MG, Myasthenia gravis; MGFA, Myasthenia Gravis Foundation of America; MMS, minimal manifestation status; PIS, post-intervention status; PR, pharmacologic remission; Pre, prednisone; RNS, repetitive nerve stimulation.
